# Case Report: Treatment of ultra-late phrenic nerve stimulation after cardiac resynchronization therapy with double-layer spacer isolation technique

**DOI:** 10.3389/fcvm.2026.1762109

**Published:** 2026-03-17

**Authors:** Cai He, Wei Wang, Yue Bao, Hongwei Han

**Affiliations:** Department of Cardiology, Wuhan Asia Heart Hospital, Wuhan, China

**Keywords:** cardiac resynchronization therapy, diaphragm contraction, dilated cardiomyopathy, isolation technique, phrenic nerve stimulation

## Abstract

Ultra-late phrenic nerve stimulation (PNS) without evidence of lead dislodgement after cardiac resynchronization therapy (CRT) can be frequently solved by reprogramming and seldom leads to reintervention. We reported a case of super-response to CRT patient, PNS occurred after twelve years of the surgery. Reprogramming the parameters was ineffective. We innovatively used a double-layer spacer isolation technique to effectively isolate the left ventricular lead and the phrenic nerve, achieving the effect of cardiac resynchronization therapy.

## Introduction

Phrenic nerve stimulation (PNS) after cardiac resynchronization therapy (CRT) is a common postoperative complication, usually occurring within 3 months after implantation and is related to lead dislodgement (1, 2). The research data on late PNS (≥3 months after implant) is relatively limited, with an incidence of approximately 13.2% (3). The incidence of ultra-late PNS (≥10 years after implant) has hardly been reported. Late PNS is more common in super-response to CRT patients, which is related to the change in the anatomical relationship between the left phrenic nerve and the coronary vein (4, 5). This PNS can usually be resolved by reprogramming and adjusting parameters. In this case, our patient's condition was not improved by reprogramming the parameters. We innovatively employed the double-layer spacer isolation technique, successfully isolating the left ventricular lead from the phrenic nerve, ensuring the isolation effect without affecting the cardiac diastolic function, and achieving the goal of cardiac resynchronization therapy.

## Case report

**Figure 1:**
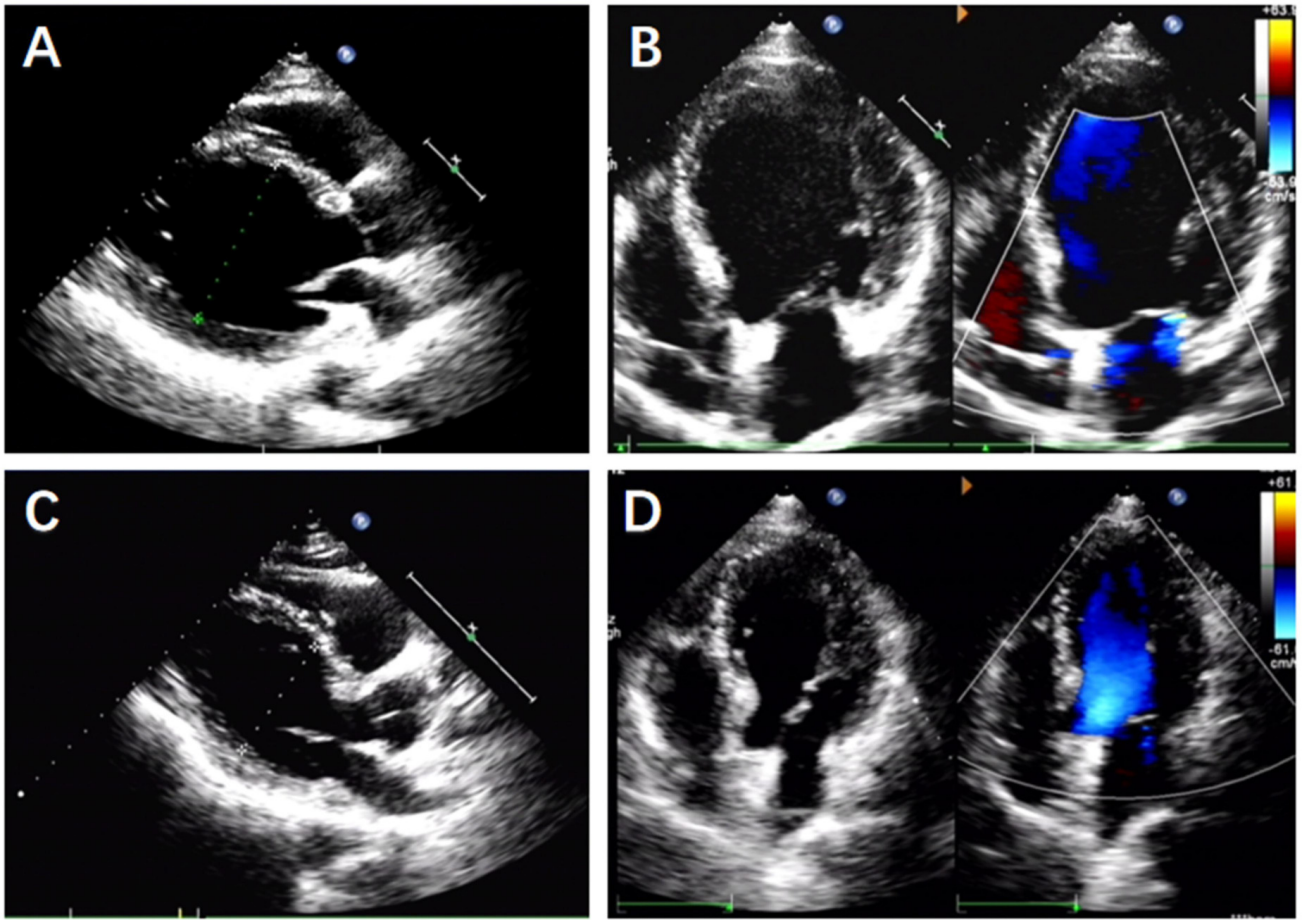
Echocardiogram. **(A)** In April 2012 chest parasternal view, left ventricular anterior-posterior diameter 7.1 cm. **(B)** In April 2012 apical five-chamber view. **(C)** In April 2013 chest parasternal view, left ventricular anterior-posterior diameter 5.4 cm. **(D)** In April 2013 apical five-chamber view.

**Figure 2:**
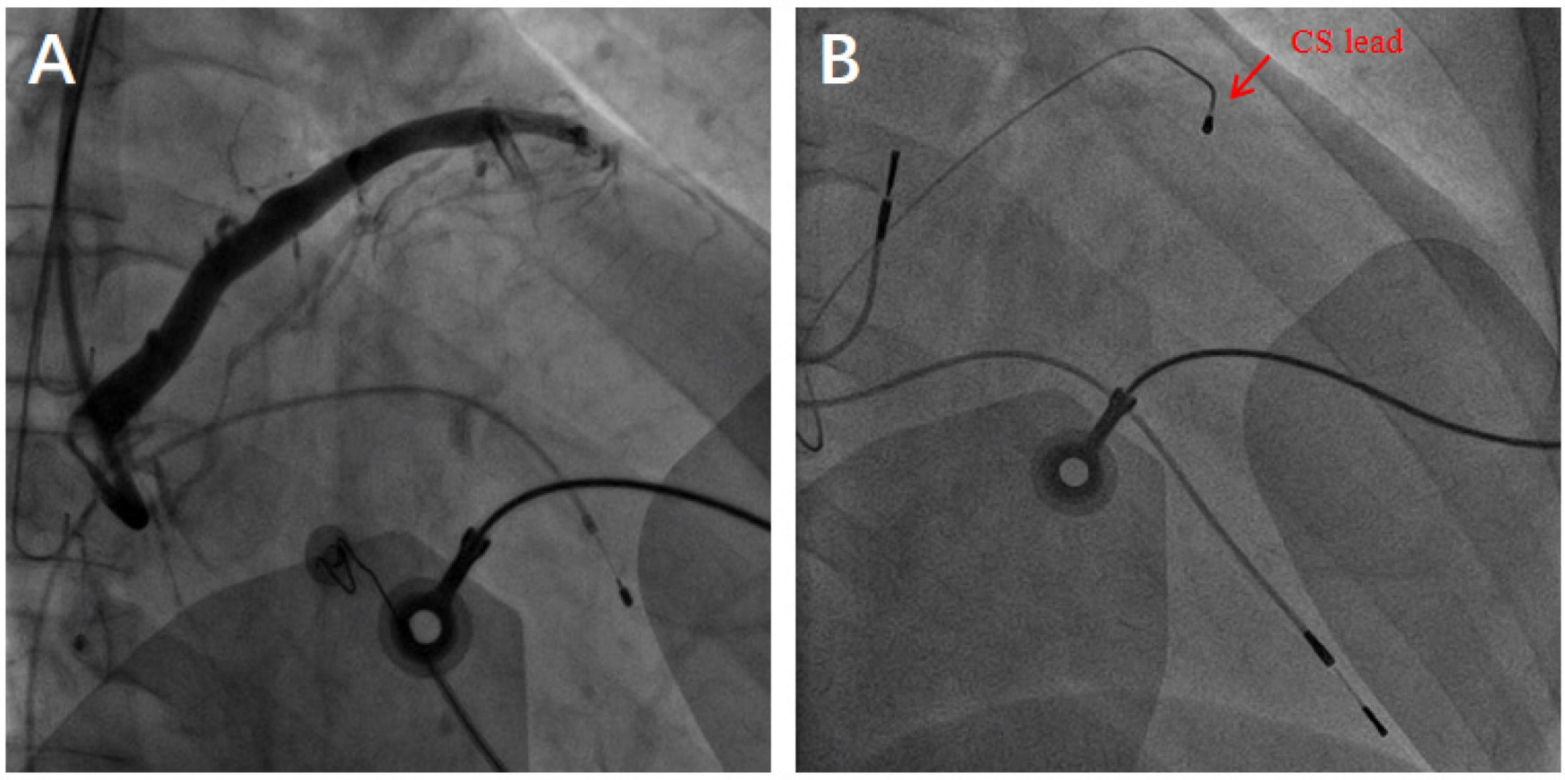
**(A)** Coronary sinus venogram, **(B)** Arrow indicates the position of the coronary sinus lead.

**Figure 3:**
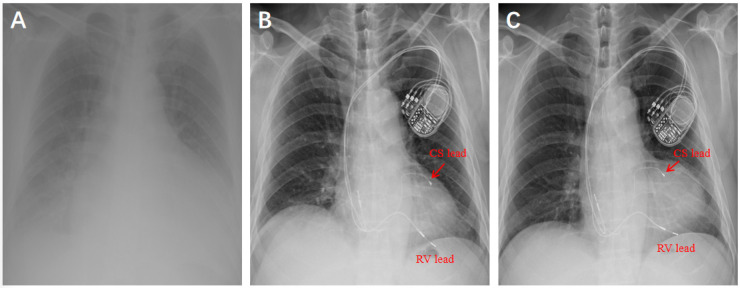
Posteroanterior chest radiograph. **(A)** In April 2012, Enlarged heart, pulmonary congestion. **(B)** In April 2013 at 1-year after CRT implatation, arrow indicates the position of the coronary sinus (CS) lead. **(C)** January 2025 at the 1-month follow-up of the surgery, arrow indicates the position of the coronary sinus lead.

A 71-year-old male patient was diagnosed with “dilated cardiomyopathy and complete left bundle branch block” in April 2012. Transthoracic echocardiography (TTE):left ventricular end-diastolic dimension (LVEDd) of 71 mm, left ventricular ejection fraction(LVEF) of 33%,generalized reduction in ventricular wall motion (Figures 1A, B). The patient underwent a cardiac resynchronization therapy (Medtronic CRT-P C2TR01) at our hospital. During the operation, it was found that the posterior side vein of the left ventricle was tortuous, with proximal stenosis and high tension, making it difficult to implant the catheter (Figure 2A). Therefore, the anterior side vein of the left ventricle was selected as the target vessel, and the lead was implanted and fixed (Figure 2B). One year after the operation, re-examination of TTE: LVEDd of 54 mm, LVEF of 48% (Figures 1C, D). Chest x-ray showed that the heart had returned to its normal size (Figure 3).

In July 2022, due to depletion of the pacemaker battery a CRT replacement surgery (Medtronic pacemaker CRT-P C2TR01) was performed. Since April 2024, the patient frequently experiences hiccups, which are unbearable. The pacemaker programming has indicated that all polarities are causing PNS. The left ventricular threshold is 0.75 V/1.5MS, 1.0 V/1.0MS. After adjusting the parameters through pacemaker programming, reducing the output and increasing the pulse width still failed to achieve satisfactory results. With the lowest output pacing, the ventricles and diaphragm were still paced intermittently. The patient experienced diaphragm contractions in the supine position, lateral position, and sitting position. Therefore, the CRT was turned off. In December 2024, TTE:LVEDd of 59 mm, LVEF of 40%, the left ventricular systolic function was reduced.

Considering the patient's coronary vein stenosis and tortuosity, it was difficult to implant the electrode lead. The lead replacement surgery was operationally challenging and might not reach the ideal location. The pacing parameters were unstable and unable to achieve the goal of cardiac resynchronization. After discussion among the surgeons and physicians, it was recommended to perform a surgical thoracotomy exploration and spacer insertion.

**Figure 4:**
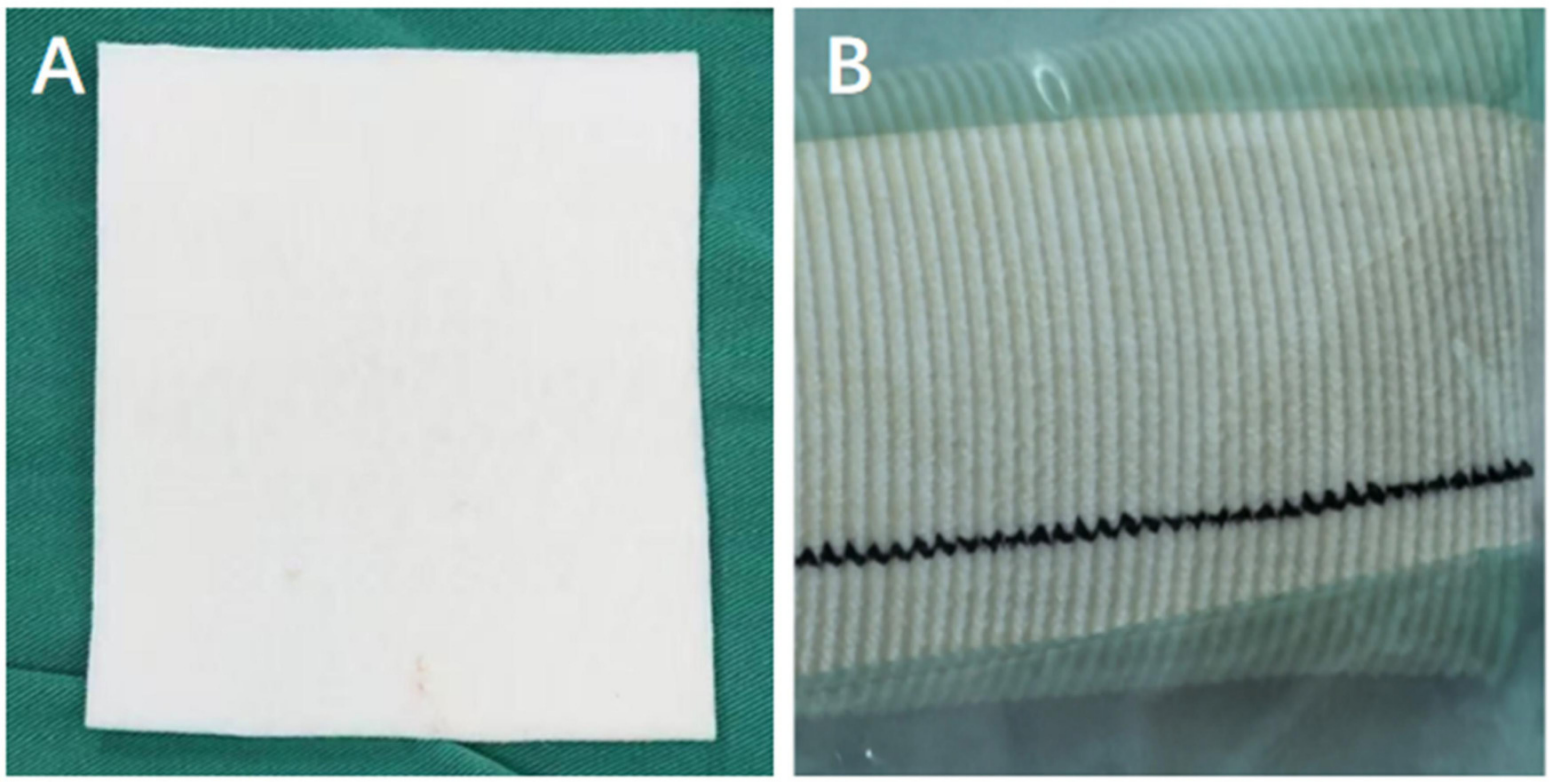
**(A)** The felt pad (polyester heart repair material, Shanghai Cheste). **(B)** The double-velvet braided artificial blood vessel (from Maquet, Germany).

**Figure 5:**
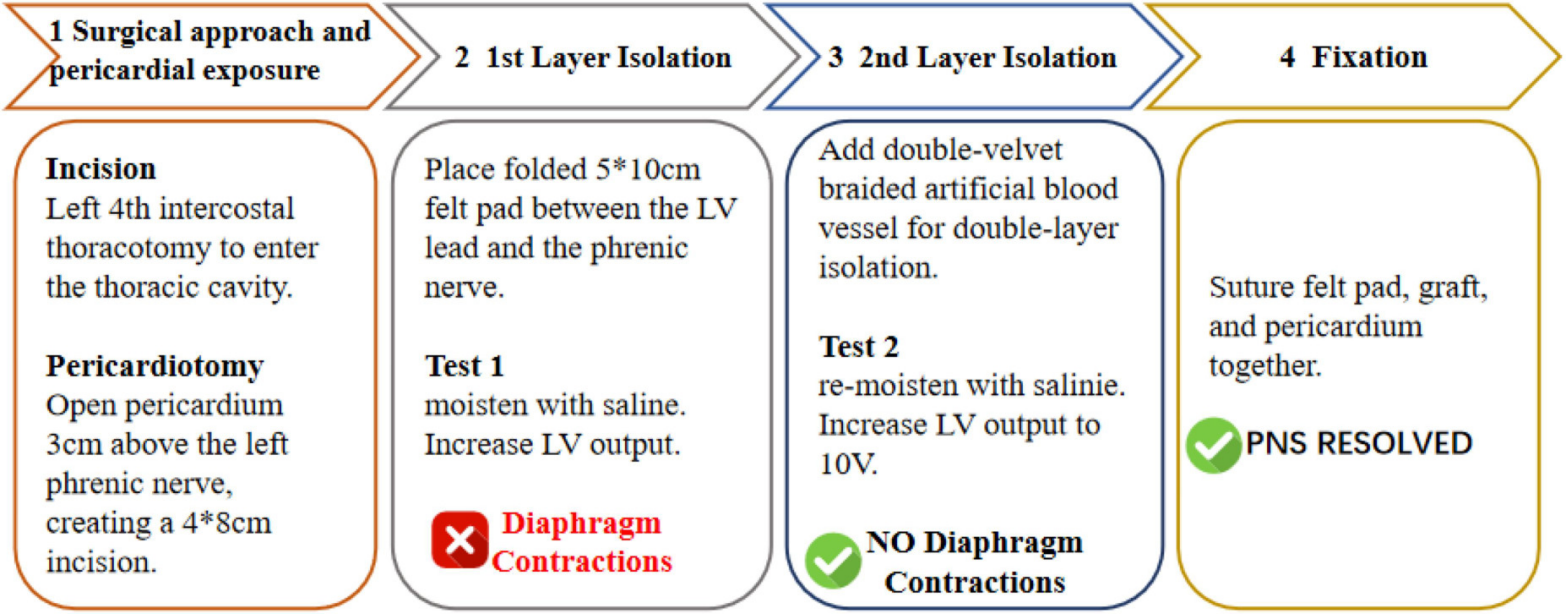
Flowchart of the surgical procedure in December 2024.

In December 2024, a thoracotomy under general anesthesia was performed. A left fourth intercostal incision was made to enter the left thoracic cavity. The pericardium was opened approximately 3 cm above the left phrenic nerve, creating a 4*8 cm incision. A folded felt pad (polyester heart repair material, Shanghai Cheste) 5*10 cm was used to isolate the left ventricular pacing lead and the left phrenic nerve (Figure 4). Inject approximately 5 mL of sterile saline at the site of the cardiac patch to fully moisten the felt patch. The programmed left ventricular output was increased, and intermittent diaphragmatic contractions occurred. Subsequently, the double-velvet braided artificial blood vessel (from Maquet, Germany) were added and overlapped with the felt sheets for isolation. Sterile saline was injected again to fully moisten the double-layer material. Left ventricular pacing output was increased to 10 V, without induction of diaphragmatic contraction. The felt pa, artificial blood vessel, and pericardium were then sutured and fixed (Figure 5).

**Figure 6:**
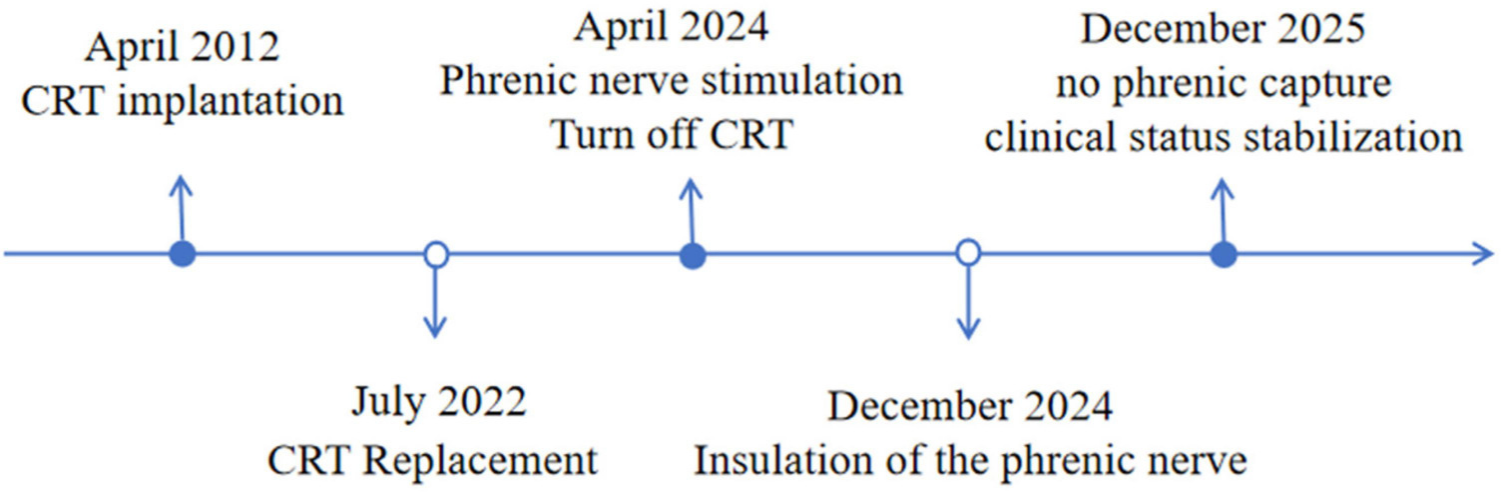
timeline with relevant data from the episode of care.

No complications occurred after the surgery. At the 1-month follow-up, the PNS disappeared, and the left ventricular pacing threshold remained fine. During the 6-month and 1-year the left ventricular pacing threshold was 1.0 V/1.5 ms and 2.0 V/1.0 ms. There was no PNS, and the cardiac ultrasound indicated no enlargement of the atrioventricular chambers and normal left ventricular contraction function (Figure 6). The successful implementation of cardiac resynchronization therapy and stable clinical condition were achieved.

## Discussion

The current treatment options for PNS after CRT include non-invasive and invasive approaches (6). The non-invasive options include reducing the left ventricular pacing output or the left ventricular threshold, extending the pulse duration to 1.5 ms, and repositioning the bipolar or quadripolar leads (7, 8). The invasive options include surgical reimplantation of the LV electrodes and isolation of the phrenic nerve through pericardial patches.

This patient underwent CRT implantation and replacement surgery. Due to reverse cardiac remodeling and local structural changes such as mild myocardial fibrosis, ultra-late PNS occurred. The CRT exhibited significant efficacy. Studies have shown that in patients with quadripolar leads, almost complete elimination of PNS can be achieved through reprogramming (7). In this case, we repeatedly adjusted the parameters, attempting to reduce the ventricular pacing output to 0.75 V and increase the pulse duration to 1.5 ms, but diaphragm stimulation was still observed. After shutting down the CRT, the heart gradually expanded and its contraction function declined. The coronary sinus (CS) lead has been implanted for 12 years and it is difficult to adjust its position. Considering that the patient's blood vessels were narrowed and tortuous when the CRT was first implanted, the operation to re-implant the electrode lead is difficult and may fail, and the risk of infection is increased. The patient has an overreaction to CRT. Currently, the programmed parameters are good. If the pacing of the conduction system cannot reach the ideal location, the goal of cardiac resynchronization cannot be achieved, and the purpose of improving cardiac function cannot be reached. Previously, surgical patching has been effective in treating such patients (2). In conclusion, we chose to use an implanted surgical patch to isolate the left ventricular electrode and the phrenic nerve.

Recently, there have been research reports on PNS due to pacemaker lead displacement (2). Chest x-rays are valuable in demonstrating clinically significant pacemaker complications. Comparing chest x-rays of the patients in April 2013 at 1-year after CRT implatation (Figure 3B) and January 2025 at the 1-month follow-up of the surgery (Figure 3C), it is observed that the position of the RV electrode has changed. The causes of pacemaker lead displacement include Direct trauma over the system, Reel's Syndrome, Twiddler's Syndrom, Intense respiratory therapy and rare factors as Reverse Ratchet’ Syndrome, tension pneumothorax (9–11). The results of our pacemaker programming indicates that the RV electrode's pacing and sensing functions are good, excluding the factor of electrode displacement. It is considered that the possible factors are myocardial remodeling and different respiratory phases, which cause changes in the rotation degree and spatial position of the heart within the thoracic cavity.

In the past, isolation of the phrenic nerve and the left ventricular lead was mostly using Polytetrafluoroethylene (PTFE) (12, 13). The study's failure to achieve electrical isolation when using felt material may be attributed to the humidification of the felt by the pericardial effusion (12). Mediratta et al. (14) used materials such as surgicel and bovine pericardial patch to isolate the LV lead and phrenic nerve, while in our surgery, we used PET material felt sheets and double-velvet braided artificial blood vessels for isolation. The material we used is polyethyleneterephthalate (PET) fiber, which is commonly used for repairing atrial septal defects and ventricular septal defects. Compared to PTFE, PET has better flexibility and breathability. It can adapt to the movement and deformation of the heart without restricting its normal functions. Research indicates that PET demonstrates superior endothelial cell retention compared to PTFE and pericardium under shear stress conditions (15). At the same time, its excellent biocompatibility can reduce tissue rejection reactions and lower the occurrence of inflammatory responses and fibrotic adhesion. The double-velvet braided artificial blood vessel is made of double-filament woven polyester containing bovine collagen and glycerol. It combines flexibility and support, stably maintaining the position of the physical barrier, blocking abnormal current conduction, and more effectively isolating the lead from the phrenic nerve. The compliance of the artificial blood vessel can be regulated by adjusting the ratio of the inner and outer layer thicknesses (16, 17). The double-layer spacer isolation technique can further enhance the isolation effect and effectively prevent the interference of electrical conduction on the phrenic nerve. The use of double-layer materials has enhanced the mechanical barrier performance and ensured long-term stability.

We innovatively employed the double-layer spacer isolation technique, successfully isolating the left ventricular lead from the phrenic nerve, ensuring the isolation effect, providing a safe and effective solution for the PNS caused by the left ventricular pacing lead. The case in this manuscript is a different independent patient compared to those in related studies and another manuscript (ID 1700683), with no data overlap or content repetition. Although the treatment techniques are similar, the patients and clinical characteristics are all different, which can provide effective references for the clinical management of phrenic nerve stimulation.

However, it still has limitations. The dual-layer combination may restrict the diastolic function of the heart, and further long-term follow-up observation is required.

## Data Availability

The raw data supporting the conclusions of this article will be made available by the authors, without undue reservation.
